# Acceptance rate of clinical study endpoints and adequacy of source documentation: experience from clinical study endpoint review in NEAT001/ANRS143

**DOI:** 10.7448/IAS.17.4.19572

**Published:** 2014-11-02

**Authors:** Juan Berenguer, Ferdinand Wit, Per O Jansson, Christine Schwimmer, Justyna D Kowalska, Juliette Saillard, Alpha Diallo, Anton L Pozniak, François Raffi, Jesper Grarup

**Affiliations:** 1 Hospital General Universitario Gregorio Marañon, ID, Madrid, Spain; 2 Department of Global Health, Amsterdam Institute for Global Health and Development, AMC, Amsterdam, Netherlands; 3 CHIP, Department of Infectious Disease and Rheumatology, Rigshospitalet, University of Copenhagen, Copenhagen, Denmark; 4 INSERM, U897, Bordeaux, France; 5 Wojewodzki Szpital Zakazny, ID, Warszawa, Poland; 6 ANRS, Service de recherches cliniques et thérapeutiques, Paris, France; 7 Chelsea and Westminster Hospital, NHS Foundation Trust, ID, London, UK; 8 University Hospital, ID, Nantes, France

## Abstract

**Introduction:**

NEAT001/ANRS143 was an open-label, randomized, non-inferiority study comparing raltegravir+darunavir/r(RGV+DRV/r) vs. tenofovir/emtricitabine+darunavir/r (TDF/FTC+DRV/r) in HIV-infected antiretroviral naïve adults. Primary efficacy outcome was a composite of virological and clinical events by week 96.

**Materials and Methods:**

Clinical trial units collected and translated supporting documentation (SD) related to the investigator-reported events. A coordinator checked events and SD for consistency and completeness. The Endpoint Review Committee (ERC) determined if clinical events met pre-defined diagnostic criteria in categories “confirmed” or “probable”. The ERC of 12 experienced, independent clinicians served in groups of three conducting individual reviews in writing, blinded to treatment arm. Differences of opinion were adjudicated in a second review by direct dialogue between reviewers. “Confirmed” events required adequate SD like laboratory, radiographic or pathology diagnostic reports. “Probable” events were typically based on clinical criteria.

**Results:**

Of the 164 serious and 3,964 adverse events reported in the study, 133 qualified for endpoint review, for a total of 153 adjudications:

Sixty of 111 per protocol endpoints were confirmed (n=53) or probable (n=7), which equals an acceptance rate of 54%. In two confirmed cases, SD was partly adequate and evaluation uncertain. Of 51 rejected events, 13 had insufficient SD, two were recurrent events. The rate of rejected events was comparable between treatments with 41% rejected events in the RGV+DRV/r arm compared to 52% in the TDF/FTC+DRV/r arm. The IRIS acceptance rate was low (3 of 18), demonstrating the difference in perception of IRIS in daily patient management and the stricter protocol definition.

**Conclusions:**

Blinded endpoint review prevented unacceptably high false positive event rates documenting that real-time ascertainment of clinical endpoints is crucial for appropriateness of the overall results. Non-confirmed events jeopardize the statistical power in this and probably all kinds of clinical studies. The rejection rate was not indicative of poor study conduct – on the contrary over-reporting prevented missing events, which would have adversely impacted the trial. Adequacy of SD and investigator training on possible differences in event criteria in daily pragmatic clinical management compared to protocol defined criteria is essential.

**Table 1:** Endpoint review results by type of endpoint

	Clinical study endpoint	Clinical study endpoint	Clinical study endpoint	Clinical study endpoint	Clinical study endpoint	Clinical study endpoint	Rashes
	
	All	AIDS	Serious non-AIDS	IRIS	Clinical Grade 4 AE	Death	Rash
Total reviewed events	111	24	35	18	29	5	42
Per Protocol Endpoints (confirmed + probable)	60	11	18	3	23	5	
Confirmed Events (for rashes: grade 2-4)	53	11	16	2	19	5	28
SD* adequate	42	10	11	1	16	4	6
SD* sufficiently/only partly adequate	10/1	1/0	5/0	0/1	3/0	1/0	19/3
Probable Events	7	0	2	15	4	0	-
SD* sufficiently/ only partly adequate	6/1	0/0	2/0	2	4/0	0/0	-/-
Rejected Events (for rashes: grade 1 or not a rash)	51	13	17	15	6	0	14
Not fulfil criteria, SD* adequate	17	3	9	2	3	0	4
Not fulfil criteria, SD* sufficiently adequate	21	8	3	8	2	0	5
Not fulfil criteria, SD* only partly adequate	13	2	5	5	1	0	5

